# OPINION: History and Perspectives of ASICs

**DOI:** 10.1016/j.bbadva.2023.100087

**Published:** 2023-03-16

**Authors:** Oleg Krishtal

**Affiliations:** Bogomoletz Institute of Physiology, National Academy of Sciences of Ukraine, 4 Bogomoletz street, 01024, Kyiv, Ukraine

**Keywords:** calcium signaling, intracellular perfusion, ASICs, P2X

## Abstract

The author recalls several particularly memorable events during his scientific career that led to the discovery of acid-sensing ion channels and ionotropic purinergic receptors. The readers learn of the events of 1975 when the first intracellular perfusion of the neuronal soma has been achieved- the event that led to the precise measurement of the calcium currents through the neuronal plasma membrane. Next, 1980 brings us to the functional discovery of the neuronal proton receptors found in mammalian sensory neurons. The molecular identity of these receptors was discovered in the lab of Dr. M. Lazdunsky and they were named acid-sensing ion channels or ASICs. Now it is clear that every mammalian neuron expresses at least one member of the ASICs family. And yet, ASICs are known for their functional diversity which is currently being studied extensively due to their prominence as pharmacological targets. Eventually, readers learn of the events of 1983 and the functional discovery of ionotropic purinergic receptors, and their molecular identification in the lab of Dr. R.A. North that coined the name of P2X ionotropic receptors.

## The foundation of Ukrainian Neuroscience research: Professor Platon Kostyuk

Independence of Ukraine in 1991 was a monumental event for the nation: it strengthened its cultural and national identity, revitalized its political movement, and led to the transformation of Ukrainian science. The Soviet-Ukrainian science was a substantial piece inherited from the Soviet imperial science, which represented military oriented nation. Correspondingly, biological sciences perceived with lesser importance, were placed at the periphery of the centralized funding. Only the unavoidable needs of the agriculture and healthcare kept Ukrainian biological sciences afloat. At the end of 1950s the official stance had softened and the Soviet science, though as military-oriented as before, became less rigid and repressing. Imperial mentality found its realization in the global ambition of possessing all branches of science. Cybernetics stopped being referred to as "the maid of bourgeoisie" and the foundation of genetics and its transfer of genetic information through genes and DNA was no longer a capital heresy. At these times the global science became interested in bioelectricity. Emerging advances in electrical engineering allowed scientists to build amplifiers and oscilloscopes to measure and amplify electrical signals produced by the biological systems; and microelectrodes to make contacts with living cells were invented. These inventions opened the door into the spectacular and dynamic world of electrical events taking place in the living organisms. This was followed by generations of young physicists, chemists and mathematicians who found these emerging fields attractive to work in. The author of these lines was one of them. In 1968, I have graduated from the department of physics at Shevchenko University in Kyiv, now known as National Taras Shevchenko University. Coming from the family of biologists, I intended to work in the basic science, and consequently applied for a position in the laboratory of Prof. Platon Kostyuk who was at that time already a legendary figure perceived by a younger generation as someone from the big wild world of top-notch science outside of the realm of the Soviet empire. Dr. Kostyuk was one of the very few people allowed to cross the Iron Curtain, as he was invited to do an internship in the lab of Dr. John Eccles, the famous Nobel Prize laureate, and a pioneer studying ionotopic mechanisms of synaptic transmission. Upon his return to Kyiv, Kostyuk was given a laboratory at the Bogomoletz Institute of Physiology of the National Academy of Sciences of Ukraine. I was the first physicist in the Kostyuk's lab and so had to break the ice of distrust. It appeared to be not so difficult because my training included electronic engineering as all physicists graduating from Kyiv University were getting a second, military profession. And at that moment such know-how appeared to be in high demand. I started with organizing voltage-clamp studies by using microelectrodes impaled into the neurons of a giant mollusk (snail). Our self-made electrophysiological rig was the first voltage-clamp facility in the entire Soviet Union. I made my amplifiers from the parts of decommissioned radar station provided to the Institute of Physiology by the Soviet Army. The voltage-clamp method allows for the precise measurement of ion currents through the plasma membrane of the living cell. This method was used by Hodgkin and Huxley to describe the events underlying the action potential generation in the giant axon of a squid. The task suggested to me by Prof. Kostyuk was to do the same but for the membrane of neuronal body, the soma. Preliminary data hinted that the properties of neuronal soma could be much different.

It appeared to be impossible to carry out thi task using available experimental approaches. There were two major obstacles: (1) it was impossible to ensure adequate voltage-clamp (so called “space clamp” condition could not be fulfilled), and (2) it was impossible to separate currents flowing into the cell (i.e. inward currents) from the currents leaving the cell (i.e. outward). In all probability the number of "players" in the somatic nerve impulse generation was larger than 2 (Na and K) as in nerve fibers.

## "The Calcium problem”

Based on the preliminary data from various sources, Prof. Kostyuk suspected that Ca2+ current was one of the potential players in the somatic membrane. The concept of calcium signaling just started to gain recognition during these years and the stakes in this game were extremely high. But let's us stop for a second and look at the working brain with a bird' eye view, as with our current knowledge. Billions of neurons generate the hundreds of billions of action potentials every second. The information is encoded in the number of such pulses and in their frequency in the train. The main feature of every train is its address specificity: where it was generated and where it goes. The qualitative property of the brain tissue is so-called use-dependence of inter-neuronal connectivity, which implies that the efficiency of the connections depends on the properties of incoming impulse trains. The existing uncontested paradigm indicates that the traces of electric activity are "written down" by the calcium. In fact, calcium ions play a role of "second messengers": they enter the cell following the nerve impulse, after which they initiate the variety of biochemical processes. As a result, the initial electric activity initiates the changes in underlying molecular machinery. The latter, in its turn, affects the electric activity, and the billions of such feedback loops make countless changes in the overall brain machinery. The memory and learning processes are among them. In other words, the wiring of the brain is due to its electrical activity. And without crossing the limits of the scientific truth, we can even postulate that the world is written down by the working brain. The "ink" which the brain uses in its writing are calcium ions. Though this picture was not that clear in the beginning of 1970th, the corresponding suspicions were already in the air, and hence, the task suggested by Kostyuk was of high importance. We shall also add the following features to the full panorama of the brain research during these days.

## Intracellular perfusion: Ca2+ current has been measured

At that time, we have worked with Dr. Vladimir Pidoplichko who became my longtime collaborator. The emerging method of isolating neurons, particularly their soma brought new hopes. Without having any precise plan, we decided to isolated parts of the plasma membrane from the neurons of the giant snail. If successful, this could make it possible to put the neuron into a small chamber mounted in a rig and to get rid of the microelectrodes. My current rig was a hopelessly inefficient one in which the isolated neuron (∼100-300 micrometers in diameter) was positioned in a hole made in a polyethylene plastic tube.

The connected oscilloscope has been producing a straight line with each sweep. And at that moment I noticed something unusual: a paraffin oil, which we used for securing the neuron in the hole, appeared to be melting once in contact with the parafilm, the commonly used laboratory sealing material. The mixture appeared to be especially sticky and formed long visible threads when touched by a microneedle. I used such thread to make a circle around the neuron sitting in the plastic hole. I looked at the oscilloscope screen and, suddenly, instead of the usual straight line, I observed the first true action potential which was recorded without any microelectrodes. I was alone in the room. Filled with emotions, I jumped into the hallway and cried "We have a perfusion!" The term "perfusion" related to the era of squid giant axon studies when the internal perfusion of the axon resulted in a breakthrough in the research on the electric excitability of neuronal fibers. It is worth noting that at those times the cytoplasm must be squeezed out from the giant axon to be replaced with an artificial saline. This step had a principal importance: the axon has continued generating axon potentials, even when refilled with this artificial saline. This was the ultimate argument: the property of electric excitability is the property of the plasma membrane, and not the cytoplasm. Which was an absolutely disrupting discovery as the entire international scientific library was filled with the books attributing this property to a cytoplasm alone. The authors of these books were active believers till the moment of truth came with the perfusion experiment performed with the giant axon of a squid. And now, few decades later, we were bound to make the first intracellular perfusion of the body of a nerve cell! After seeing what we saw on the oscilloscope screen, without any regrets we disassembled our microelectrode rig–so precious yesterday and so useless today–and built a new, improved one. The anticipated success was too attractive to lose any second, and our moment of truth came just in few days. The neuron was put into a hole, its membrane was sealed using this newly tested glue. The part of the cell membrane that was facing the calcium-free artificial internal solution was expected to lose its barrier properties and become permeable. The membrane facing the Ringer saline demonstrated normal ionic currents characteristic for electrically excitable neuron. We replaced potassium in the artificial internal solution and observed the disappearance of the outward current. The first important fact became immediately clear: the inward current carried by calcium had a slow kinetics which was masked by K+ currents in the cells with intact cytoplasm. And yet, when we submitted the manuscript describing this work, the *Nature* reviewers insisted that without visible mechanical destruction of the membrane we have rather a dialysis than perfusion, meaning a transfer of molecules via pores [1]. Further experiments demonstrated that the term "perfusion" was absolutely justified. Subsequent publications in the *Journal of Physiology* served as important milestone in the understanding of the electric excitation and calcium signaling in the neuronal soma [2,3].

### Single Channels

The development of ideas concerning the mechanisms of electric excitation brought the concept of ion channels originated at the plasma membrane. Development of this concept, when observed retrospectively, may serve as a triumphal example of an analytical approach. Smooth processes of recharging the cell membrane by a hypothetic molecular entity, such as the ion channels, were expected to be a superposition of all-or-nothing microscopic current steps. Many logical arguments were suggested by the inventive scientific brains to make this picture persuasive. But still, there had to be a final proof, direct observation of this phenomena. The waiting time for this discovery was not long: the laboratory of Drs Ervin Neher and Bert Suckmann in Göttingen was pursuing similar aims as we were, though they were more concentrated on their ultimate goal: the single channel recording. Our groups have friendly relationship and Ervin Neher visited us quite often. We were the first to make intracellular perfusion, but then the fortune arrived at Göttingen, which happened about two weeks before I visited Ervin there. In his account what happened, there was also a heuristic moment of surprise. Methodically we were on the same track, but with one, as if not minor, difference: the pores to be plugged by the neuronal membrane were made in Germany from glass, while here in Kyiv we used plastic. The contact with the cell seemed to be plugged by the glass pore ideally and no one needed the microelectrodes anymore. Eureka moment came when Erwin increased the gain on the amplifier and found peculiar steps of current passing through this "plugged" tip of the electrode. These were the first ever recorded signals from a single ion channels, "the quanta of electrical excitation".

### "Receptor for protons"

One cannot be a loser all the time. So quite soon we got something else to play with. In a couple of years after the described events we were doing experiments in Kyiv studying the properties of the sensory neurons (the cells from the dorsal root ganglia). We applied our intracellular perfusion method to the neurons isolated from the spinal ganglia of a rat. The experiment seemed to be compromised: some neurons demonstrated a strange current: it was slowly activating and inactivating. Its amplitude varied significantly from sample to sample, but its dependence was stochastic and did not correlate with the concentration of any known added substance or ions. This phenomenon was coined an "artifact", but had to find what was wrong. The answer as often happens was on the surface: our stock solution to make working saline was not pH buffered so that resulting pH of any consequent samples could attain an arbitrary value. Strange current was activated when the cell was submerged into particularly acidic saline. The value of this current was rather small. But the slow kinetics of its activation appeared to be determined by the rate with which we could change the solution in the experimental chamber. This implied that if such rate would be further reduced, we should see no current at all: the process of its decline would prevail. This situation was analogous to the measurements on the rapid voltage-gated ion channels. To perform proper measurements of their activity one had to apply changes in the activating parameter – the voltage - much faster than the reaction of the channels to this change. It became immediately clear that for the studies of rapid membrane chemosensitivity one needs the achieve the "concentration clamp" which is a similar approach to the mentioned above voltage clamp.

**Fig. 1:**
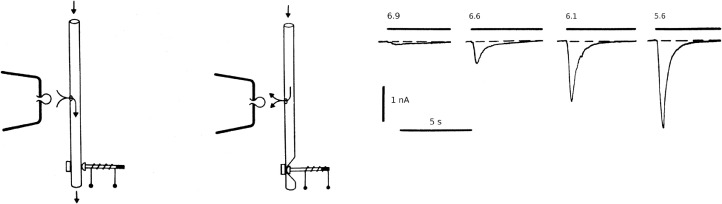
The first observation of the proton-induced currents. Left panel: schematic diagram explaining the method of rapid replacement of the external solution. The cell is placed in the pore for internal perfusion. The external solution #1 is applied to the cell surface. Closing the valve results in the flowing of the external solution #2 from the tube to the cell. Right panel: H+-induced currents observed after the rapid changes of the external pH from 7.4 to the levels indicated above the curves. Adapted from [5].

Recalling that strange night, I feel that it was probably a kind of hunting drive that pinned us to the lab till the morning. It was impossible to postpone a possibility of seeing mysterious phenomenon in its true shape. A very simple idea allowing us to change the external solution in the microvolume within a several milliseconds was put into life just within a couple of hours. The moment of truth finally occurred when a slightly acidic saline (now properly buffered) was puffed at a neuron changing the pH over its entire surface within 10 mseconds. And this is when we observed a current (Fig. 1), which was large and well-comparable to the "usual" voltage-operated currents responsible for the generation of the nerve impulse. Now we could see that the activation of this current was rapid, with the rate exceeding the speed of solution exchange achieved by our newly assembled concentration clamp rig. But now we were more confident than ever: our equipment allowed to measure the correct amplitudes of the current: its decline after achieving the peak value was much slower. The principle of the concentration clamp appeared to be in demand. It was used by Neher and Sakmann in their first study on the chemically activated single channels [4]. In the meantime, by changing the holding voltage we found that the selectivity of these proton-activated channels to Na+ vs K+, i.e. Na/K was as high as the selectivity of usual Na+ channels. It appeared highly unlikely that the channels so selective could be just a part of a nonspecific leakage. The words have automatically assembled in my head: "the receptor for protons".

### The thorny path of discovery

An important reminder: we found the proton-activated Na+ conductance in the spinal ganglia of a mammal. All the neurons in these ganglia have specific sensory functions: mechano- and thermosensitivity, nociception etc.

The concept of pain sensation attracted our attention because in a great majority of clinical cases, the pain is accompanied with the acidification of the affected tissue. It seemed so seductive to imagine that "our" receptor was a major receptor of pain depolarizing sensory neurons in response to a specific pathological state. The most common situations where pain is observed are inflammation, ischemia and similar. With this working theory it is logical to imagine that the selective and effective blockers of the receptor for protons will become a magic bullet in the family of novel painkillers, which was quite limited so far. We managed to find some supportive, though indirect, evidence and published a theory formulated in the title of the article in the *"Neuroscience Letters"* [6]: "A receptor for protons in the sensory neurons of rat may participate in nociception" (the perception of pain is called *nociception* from the Latin *nocere* - harm). This publication was met with some curiosity, but certainly with more skepticism rather than the applause. The postulation of a specific receptor with the activator so non-specific and ubiquitous as protons was too much. For us this was a time of discovery, probably the last rainfall of this kind for the electrophysiology.

Returning to the bird's eye view on the brain, which is perhaps the most complex structure in the Universe known to us, it houses two classes of phenomena: the electrical and chemical. And to be even more specific, one can even attach the prefix bio in both cases. The decades between 1950 and 1980 were marked by the quick development of electronics fueled in part by the powerful advances in physics and resulted in the many breakthroughs in the understanding of the electrical side of the brain activity. One could proudly state that the mystery of major bioelectric processes ceased to exist. But this discovery opened a new level of understanding: the amazingly complex and organized machinery was functioning behind a perceived hectic electric activity within our scull. This made it especially clear how unimaginably complex was the overall design of our personal non-manmade computer. Billions of impulse trains are created by the molecules constituting the brain tissue. Information is encoded in the parameters of trains. And here is what may seem to be the main "trick" of such design: impulses created by molecules spread to the structure/function-determined addresses. It is important to note that in reaching their molecular targets, the impulses initiate changes in the molecules. The altered molecules will now change encoded information in the future trains of the nerve impulses. Some of the molecular changes stay memorized on a long-term basis. The structures responsible for this memorizing are also known these are the synapses, the contacts between the neurons. Current paradigm indicates that ourselves and all our memories are stored in the synapses. And the volume of this memory is practically unlimited. As limitless, as the field of study of the molecular machinery of the brain. Back in 1980, we could only dream of being so lucky as to find a brand-new bioelectric mechanism of considerable functional significance. Our dream was that we found a major "receptor of pain". But before attributing this role to the newly discovered "receptor for protons", we had to prove that the response to a pH decrease is happening due to its activity. A potential alternative explanation could be that some already known receptor/molecule capable of changing its conformation in response to acidification of solution is responsible for the observed effect.

Not so long after initial observation of pH-activated current, I have presented a talk on this work at a symposium that took place in Uzbekistan. It was a springtime, still not too hot, but quite sunny. Somebody called my name. It was Prof. Hans Dieter Lux, a prominent German neurophysiologist who attended the same symposium. He said something like: “I am sorry to say what I say, but it appears, that the receptor for protons in the sense you report does not exist; this is the activity of a calcium channel deprived of calcium ions. It was also your discovery - right?”

A sympathetic tone of Professor Lux indicated that I must be upset – and I was indeed. Earlier this year we published a paper demonstrating that when deprived of Ca2+ in their surrounding a normal calcium channels could pass Na+ and do this quite efficiently [3]. The idea of Lux was clear: "our" receptor starts passing Na+ when subjected to a low pH, that is to increase a concentration of protons.

Protons are potentially capable to substitute calcium ions on the binding sites and here we are: it's not a new receptor with supposedly so powerful function, but merely calcium channels converted into their sodium permeable hypostasis.

This blow was powerful. Soon Lux et al. published an extensive paper in *Journal of Physiology* [7] describing how tricky the source of experimental errors could be. While I was actively victimized, I did not believe this paper. My argument while being rock-solid was negativistic and so remained internal: every sensory neuron has calcium currents, but not all of them demonstrate pH-activated sodium currents. We investigated whether this current can also have a calcium component. And we found it in some neurons indicating that pH-activated currents were quite non-homogenous. While they allow calcium ions to enter their channel, they cannot be active due to the substitution of these ions. I have reported this at the British Physiological Society meeting, but my arguments, unfortunately, were not met with enthusiasm. The dogma in the field was strong: the ubiquitous proton cannot be a specific agonist (aka activator) of the receptors that carry out a specific (though still unknown) function. While not bowing to this dogma, and yet not being supported by the scientific community, our attention turned to the old-time approach which allowed Hallett Dale and Otto Loewi to discover one of the most functionally important neuromediators [8] and to strengthen the concept of chemical neurotransmission.

Our hypothesis was that there is some specific agonist activating "our" receptors and that we shall find it when applying homogenates of different tissues to the neurons sensitive to protons in the way we have found. We started doing these experiments and immediately encountered a mystery: many sensory neurons responded to the homogenates by the activation of an inward current. But this current had nothing to do with the receptor for protons. There were three of us doing these experiments: in addition to Dr. V. Pidoplichko and myself, our group was joined by Dr. Sergei Marchenko (who untimely passed away in 2017). I summarized our situation: "which substance is present in every tissue and is as ubiquitous as protons?" And Sergei was the first to say: "ATP". In several minutes of experimentation, we were sure, this was ATP. Due to the extraordinary research of Prof J. Burnstock group we have heard of purinergic (adrenergic) receptors. But our knowledge was so superficial that we didn't even understand that we made another discovery. Jeoff Burnstock dealt with purinergic metabotropic receptors, that is, the receptors coupled to the G-protein signaling and the one that regulates metabolic processes. We were the first to demonstrate the existence of a purinergic ionotropic receptors, that is, the receptors operating as a bona fide ion channels. Our priority was confirmed by Alan North, one of the leading specialists in the field [9]. At the meantime the hypothesis of "pain receptor" was heavily debated. We ourselves violated its harmony with the finding that many brain neurons present a receptor for protons. And many of these cells had nothing to do either with pain reception or any other primary sensation. The flow of time was converting biology into the new biology and one day it happened.

I was at the symposium in France and the speaker, Prof. Michel Lazdunski, all of a sudden said that it was his special pleasure to see me in the hall since he has good news for me: the receptor for protons exists and it has been cloned. This meant that using gene cloning approach, his lab has got a gene sequence and a molecule responsible for the receptor for protons. It earned the right to acquire a permanent name. From now on it is called Acid Sensing Ionic Channels, or ASICs [10]. The focus of uncertainty stopped being existential. Now the question was, what are the functions of this whole new family of ASICs that were cloned with the speed and efficiency characteristic for the emerging field of molecular biology. This family comprises eight subunits assembling into a combination of functional triads. Very soon the problem of functions became multiplied once more: within several next years it became clear that every mammalian neuron expresses at least one, but most probably several subtypes of ASICs. Many types of glial cells also carry these important molecules. It is already clear that the ASICs participate in the plethora of functions in the mammalian brain.

Some of them, like long-term plasticity (i.e. learning and memory), are of uttermost importance. However, not a single case has been reported so far when the role of ASICs would be critical. This conclusion is supported by the following straightforward observations: ASICs knockout experiments were never lethal [11], and yet this also may indicate a particular redundancy, and global knockout of the entire family may provide a much-needed clue. The mechanism of vital physiological importance must be dealt by pharmacologists with great caution or should not be affected at all. In this respect the plethora of ASICs subtypes growing in our neurons and capable of modulating almost any physiological function may be a robust pharmacological target.

## Data Availability

No data was used for the research described in the article.
